# UV-Induced Radical Photo-Polymerization: A Smart Tool for Preparing Polymer Electrolyte Membranes for Energy Storage Devices

**DOI:** 10.3390/membranes2040687

**Published:** 2012-10-17

**Authors:** Jijeesh R. Nair, Annalisa Chiappone, Matteo Destro, Lara Jabbour, Giuseppina Meligrana, Claudio Gerbaldi

**Affiliations:** 1 Department of Applied Science and Technology - DISAT, Politecnico di Torino, C.so Duca degli Abruzzi 24, 10129 Torino, Italy; Email: matteo.destro@polito.it (M.D.); giuseppina.meligrana@polito.it (G.M.); 2 UMR 5518 CNRS-Grenoble-INP, Domaine Universitaire, 461 rue de la Papeterie, BP 65, 38402 St. Martin d’Hères, France; Email: lara.jabbour@lgp2.grenoble-inp.fr; 3 Center for Space Human Robotics @Polito, Italian Institute of Technology, C.so Trento 21, 10129 Torino, Italy

**Keywords:** photo-polymerization, methacrylate, polymer electrolyte, cellulose hand-sheets, nanoscale microfibrillated cellulose, electrochemical characterization, lithium iron phosphate, lithium polymer battery

## Abstract

In the present work, the preparation and characterization of quasi-solid polymer electrolyte membranes based on methacrylic monomers and oligomers, with the addition of organic plasticizers and lithium salt, are described. Noticeable improvements in the mechanical properties by reinforcement with natural cellulose hand-sheets or nanoscale microfibrillated cellulose fibers are also demonstrated. The ionic conductivity of the various prepared membranes is very high, with average values approaching 10^-3^ S cm^-1^ at ambient temperature. The electrochemical stability window is wide (anodic breakdown voltages > 4.5 V *vs*. Li in all the cases) along with good cyclability in lithium cells at ambient temperature. The galvanostatic cycling tests are conducted by constructing laboratory-scale lithium cells using LiFePO_4_ as cathode and lithium metal as anode with the selected polymer electrolyte membrane as the electrolyte separator. The results obtained demonstrate that UV induced radical photo-polymerization is a well suited method for an easy and rapid preparation of easy tunable quasi-solid polymer electrolyte membranes for energy storage devices.

## 1. Introduction

Li-based battery systems, traditionally designed for the field of portable electronic devices, have undergone rapid and substantial performance improvements with the aim of becoming the system of reference in the huge market of electric vehicles (E*Vs*) and hybrid-electric vehicles (HE*Vs*) [1,2,3,4,5,6]. Although a commercial reality, these power sources are still the object of intense R&D aiming at improving their performances for their use in high-end applications, such as in the chassis of future hybrid and electric vehicles as well as in aerospace systems [7,8,9]. High performing innovative materials are important for all the components of a Li-ion cell. The electrode materials need to have high capacity and durability, while the electrolyte should be a solid membrane capable of high ionic conductivity even at ambient temperature, with good mechanical and interfacial properties and stable performances. In all cases, the materials must be low cost, environmentally friendly and with high safety standards, beyond high specific performance, which are key-factors in the transportation field.

In the last few years, the electrolyte component has undergone a complete transformation from all-liquid to all-solid [6,10,11] and/or gel-like [12,13]. Two classes of materials have been primarily used as polymer electrolyte: solvent-free membranes, formed by blending a thermoplastic polymer with a lithium salt and gel membranes formed by thermoplastic polymers trapping the liquid solution of the electrolyte. Typically, the solvent-free membrane is based on poly(ethylene oxide) and still suffers from poor ionic conductivity at ambient temperature [6,14,15,16]. The gel membrane is usually made of poly(vinylidene fluoride) [17,18], its preparation requires a long mixing and drying time to form a free-standing film and, once obtained, it can dissolve in the same swelling solvent, especially if the temperature increases.

Thermo-set membranes prepared by UV-induced free-radical photo-polymerization technique could be an interesting alternative to the present products as this process has excellent versatility in application. It is a well-established polymerization technique, taking place at ambient temperature under UV light [19]. The potential of this technique, commonly employed for the preparation of coatings, inks and for the production of optical and electronic devices, can be diverted to our field of interest to obtain very fast, low cost production and to have an environmentally friendly approach, as the use of solvents is almost avoided. In fact, highly cross-linked polymers are readily synthesized by irradiating an appropriate formulation of multifunctional monomers, namely acrylates and methacrylates, in the presence of a photo initiator [20,21,22].

Song and co-workers [23] studied the use of UV curing to prepare chemically and physically cross-linked PEGDA/PVdF blend gel-electrolytes of high ionic conductivity. In line with this tendency, in recent years our research group carried out a systematic investigation on those materials which appear particularly promising for the development of lithium-based batteries with improved characteristics and performances [20,24,25].

In the present article, we report the synthesis and characterization of quasi-solid polymer electrolyte (PE) membranes made from di- and mono-functional methacrylates by a rapid process of UV-induced free radical photo-polymerization. PEs were thoroughly investigated for their physico-chemical and electrochemical properties. Later, they were also reinforced by specifically modified (photo-grafted) cellulose hand-sheets or nanoscale microfibrillated cellulose fibers resulting in high mechanically stable electrolytes with good performance in Li-based cells.

## 2. Results and Discussion

### 2.1. Quasi-Solid Polymer Electrolyte Membranes by UV-induced Photo-Polymerization

The preparation of the quasi-solid polymer electrolyte membrane (namely, RC-1) involved the use of a reactive mixture prepared by mixing the materials described in paragraph 3.1 in definite proportions as described in Table 1.

**Table 1:** Exact compositions (in wt.%) of the reactive mixture used to prepare sample RC-1, along with its thermal properties.

sample	BEMA	PEGMA-475	1.5 M LiTFSI solution	*Darocur1173*	*T*_g_/°C	*TGA*/°C	
						T_10_	T_50_
RC-1	36	16	45	3	−63.8	181	368

**Figure 1:**
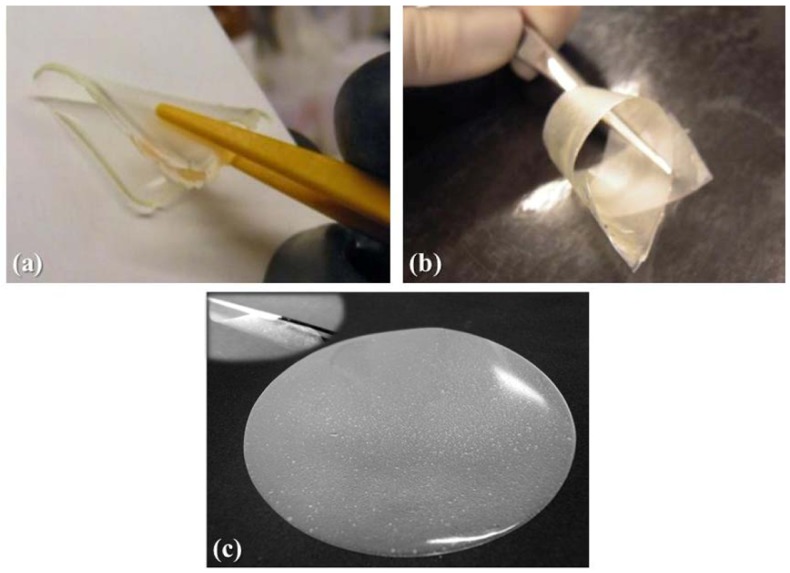
Appearance of the different quasi-solid polymer electrolyte membranes prepared: (**a**) RC-1 obtained by copolymerizing the monomers BEMA and PEGMA-475 via UV irradiation with the *in-situ* addition of a 1.5 M LiTFSI electrolyte solution; (**b**) modified-cellulose handsheet reinforced MC-PE polymer electrolyte membrane; and (**c**) microfibrillated cellulose reinforced MFC-PE polymer electrolyte membrane.

The polymer electrolyte membrane RC-1, obtained by copolymerizing the monomers BEMA and PEGMA-475 with the *in-situ* addition of the lithium salt and the electrolyte solution on exposure to UV irradiation, is a transparent, freestanding, extremely flexible and non-sticky membrane, as shown in Figure 1a.

The percentage of double bonds (>C=C<) conversion during UV exposure was evaluated from kinetic studies using real-time FT-IR technique. Results obtained showed that the reactivity of the monomers mixtures was in an acceptable range and a quantitative yield was obtained within a few seconds. In fact, the total conversion of reactive ingredients of RC-1 into products was around 63% and the respective maximum conversion was reached in less than 120 sec. A prolonged UV exposure time did not modify the total conversion. As already demonstrated [19], a 180 sec time of irradiation was sufficient to achieve the maximum conversion.

**Figure 2:**
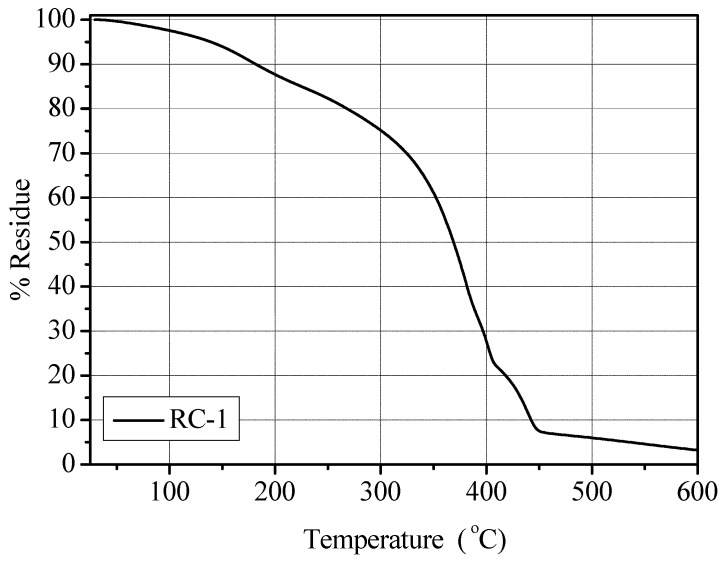
TGA analysis under N_2_ flux (temperature range 25–600 °C) for the RC-1 polymer electrolyte membrane.

The polymer membrane RC-1 showed a *T_g_* of −63.8 °C. Though the *T_g_* value was found to be very low, the obtained PE was still self-standing, extremely flexible and easy to handle. The thermal stability was assessed by thermo-gravimetric analysis under flowing nitrogen in a temperature range of between 25 and 600 °C. The results obtained demonstrated that the thermal stability of a pristine polymer membrane (*i.e*., not containing the liquid electrolyte solution) was high, up to 300 °C [20]. The EC-DEC components of the liquid electrolyte restricted the thermal stability up to approximate 120 °C, when the solvents began to evaporate (in fact, a weight loss of approximate 5% occurred at about 150 °C). However, these results indicate that the obtained PE can be safely used up to 100 °C, still well within the limit for application as electrolyte in Li-based batteries. The TGA thermo-gram of the RC-1 polymer membrane is given in Figure 2. It shows a T_10_ value (*i.e.*, the temperature at which 10 wt.% of the sample is decomposed) of 181 °C and T_50_ value (*i.e.*, the temperature at which 50 wt.% of the sample is decomposed) of 368 °C.

**Figure 3:**
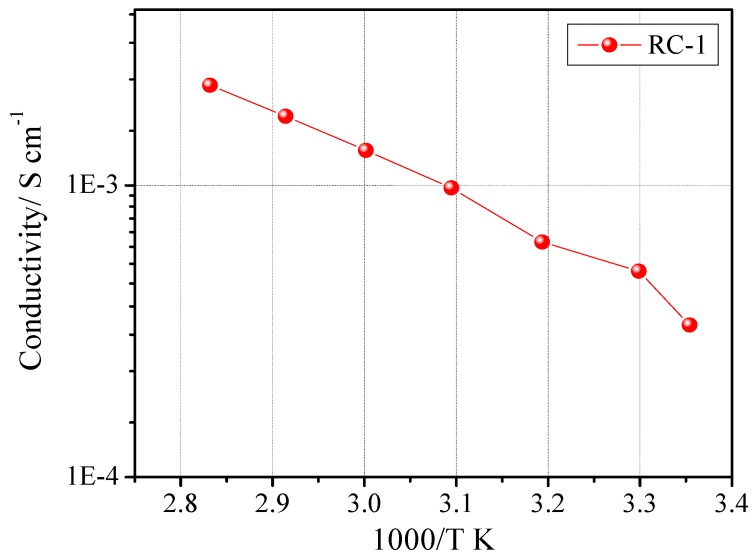
Ionic conductivity *vs*. temperature plot of sample RC-1. Data obtained by impedance spectroscopy.

The ionic conductivity of the sample was tested by constructing a symmetric cell with the SS-316/RC-1/SS-316 configuration. The Arrhenius plot for sample RC-1 is reported in Figure 3. The plot exhibited the typical Vogel-Tamman-Fulcher (VTF) curvature associated with amorphous materials [20]. The optimum ionic conductivity values were achieved by varying the components concentration and their quantity. The rapid UV-induced photo-polymerization process gives this freedom to polymer chemists/electrochemists to fine tune each and every ingredient of the final reactive mixture along with a facile synthesizing procedure. The EIS studies clearly demonstrated that high ionic conductivity values were achieved, at ambient temperature as well as at 80 °C. In fact, RC-1 showed an ambient temperature conductivity of 3.3 × 10^−4^ S cm^–1^ and 2.2 × 10^−3^ S cm^−1^ at 80 °C.

In view of its possible application as electrolyte in lithium polymer batteries, the RC-1 polymer electrolyte membrane was further tested in terms of other general electrochemical properties, such as anodic stability window and interfacial stability *vs.* the lithium metal electrode. The electrochemical stability at potential values anodic with respect to lithium at a scan rate of 0.100 mV sec^-1^ was evaluated at ambient temperature. The current-voltage curve was obtained for a working acetylene black electrode swept in a cell using RC-1 as separator and a Li metal counter electrode. The onset of the current increase, which is representative of the decomposition of the electrolyte, indicates an anodic break-down voltage of approximate 4.5 V *vs.* Li. A high decomposition potential like the one showed by RC-1 membrane is certainly welcome from a practical application viewpoint. Moreover, the anodic scan showed very low residual current observed prior to breakdown voltage, confirming the purity of the prepared PE.

**Figure 4:**
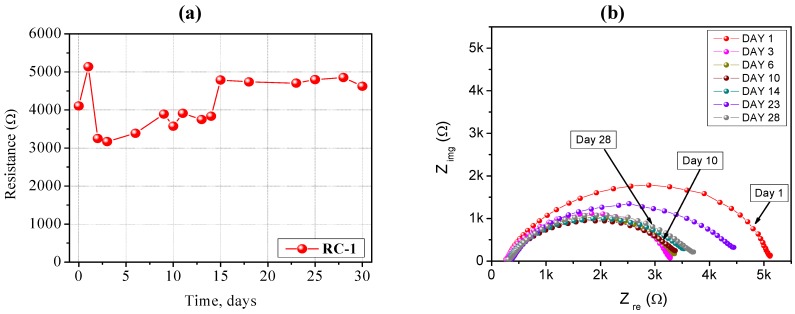
(**a**) Time evolution of the interfacial stability of a Li/RC-1/Li symmetrical cell, stored under open circuit potential conditions at ambient temperature; (**b**) Impedance spectra (Nyquist plots) of the same Li/RC-1/Li symmetrical cell. Electrode area: 0.785 cm^2^. Frequency range: 1 Hz–100 KHz.

The impedance spectra carried out on a Li/RC-1/Li symmetrical cell stored for long time periods under open circuit potential conditions at ambient temperature are shown in Figure 4a,b. It is well known that the resistance of the cell is composed of the bulk resistance (R_b_) of the electrolyte and the interfacial resistance (R_i_) which reflects the interfacial situation between the electrodes and the electrolyte. At high frequency, the intercept with the real part (Z_re_) corresponds to the bulk resistance, and this allows calculation of the ionic conductivity of the PE. This value increased only slightly with time, meaning that the liquid electrolyte embedded into the polymer network did not lose its electrochemical properties because of the non-volatile nature of the organic solvents and it showed good compatibility with the lithium metal electrode. The value of R_i_ increased quickly during the first days, indicating the formation of the passivation layer onto the surface of the Li metal electrode as a result of the reactivity with the polymer electrolyte membrane. It subsequently decayed and, finally almost stabilized at a value ~4700 Ω cm^−2^. R_i_ remained very stable for a long period of time.

**Figure 5:**
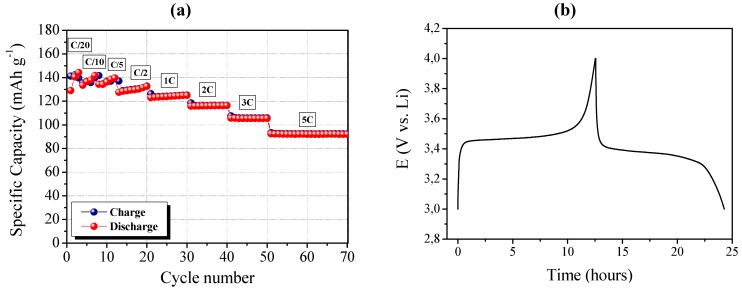
(**a**) Ambient temperature cycling performance of a LiFePO_4_/RC-1/Li polymer cell at different C-rates from C/20 to 5C (1C = 0.7 mA with respect to a LiFePO_4_ active mass of about 4 mg); (**b**) Typical charge and discharge cycle run at ambient temperature.

Finally, the RC-1 polymer electrolyte membrane was assembled in a complete lithium polymer cell laboratory prototype, and its electrochemical behavior was investigated by means of galvanostatic charge/discharge cycling. The response of the prototype, assembled by combining a lithium metal anode with a LiFePO_4_/C composite cathode and the RC-1 PE as the electrolyte separator, is reported in Figure 5. It shows the specific capacity of the cell as a function of the cycle number at ambient temperature and at different C-rates ranging from C/20 to 5C.

The cell delivered a specific discharge capacity higher than 140 mAh g^−1^ during the initial cycles, when using the low current density of C/20. As the current density increased, the specific discharge capacity decreased only slightly. Actually, at the discharge rate of 1C, the cell was able to deliver a discharge capacity of about 125 mAh g^−1^, and about 95 mAh g^−1^ at the very high discharge rate of 5C. Good performance at high current rate might be ascribed to the efficient ionic conduction in the polymer separator and the favorable interfacial charge transport between electrodes and electrolyte in the cell. Figure 5b shows a typical charge (lithium removal from LiFePO_4_ to form FePO_4_) and discharge (lithium acceptance by FePO_4_ to reconvert into LiFePO_4_) cycle run at ambient temperature. The potential drop in passing from charge to discharge was observed to be small, which means low diffusion resistance of the cell. The galvanostatic charge-discharge profiles showed very flat voltage plateaus at about 3.47 V *vs.* Li on charge and at 3.38 V *vs.* Li on discharge, reflecting the good properties of the LiFePO_4_/C cathode, namely high specific capacity and steepness of the plateau with minimum over-potential.

### 2.2. Cellulose Handsheets Reinforced Quasi-Solid Polymer Electrolyte Membranes

In the previous paragraph, we described the characteristics and performance of a quasi-solid polymer electrolyte membrane obtained by a fast UV induced free radical photo-polymerization process. Although it showed remarkable electrochemical performances, its mechanical resistance was not fully satisfying under intense mechanical abuse. Thus, we tried to improve it, especially considering the possible application as separator in thin-film flexible cells. To this purpose, incorporation of modified cellulose (MC) handsheets as a reinforcement is here proposed to improve the mechanical behavior of quasi-solid polymer electrolytes. The composition of the polymeric matrix was the same as for the previously discussed RC-1 sample and the preparation method is discussed in Paragraph 3.3. The appearance of the reinforced gel-polymer electrolyte, namely MC-PE, is shown in Figure 1b: it was a highly translucent, freestanding, extremely flexible and non-sticky membrane. 

**Figure 6:**
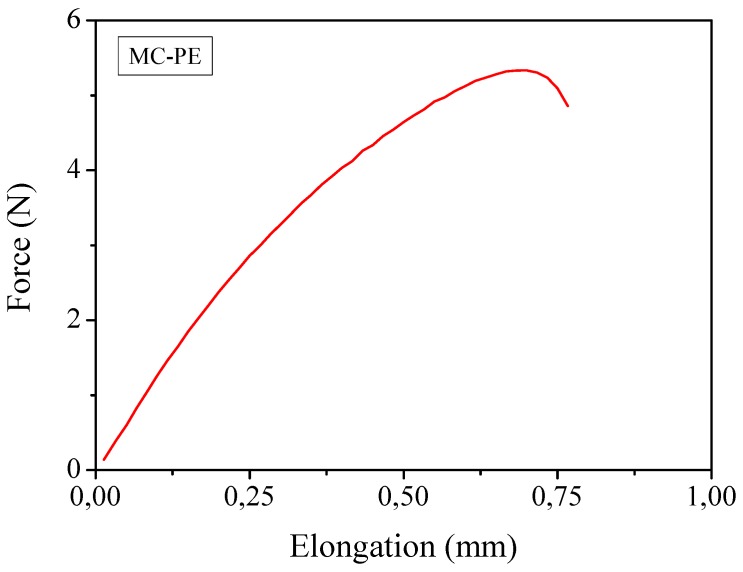
Mechanical measurements through traction test of the reinforced MC-PE at ambient temperature.

The *T*_g_ was very low indicating that at ambient temperature the polymer membrane was in the rubbery state. Mechanical properties, obtained by means of tensile test studies revealed that the average Young’s modulus was 395 MPa and the average tensile strength was 2.3 MPa. A typical force-elongation curve is reported in Figure 6. If one considers that the liquid content was calculated to be approximately 30 wt.%, these are very high values. It is important to note that, without cellulose reinforcement, the polymer electrolyte could not undergo this test. Such an excellent mechanical behavior is by all means a positive property: it may also improve the safety features of quasi-solid PE membranes, because the cellulose fibers could block the growth of lithium dendrites, as the cellulose fibers are highly crystalline and very strong.

The thermal stability of the reinforced polymeric film was assessed by thermo-gravimetric analysis under nitrogen flux. There was a distinct weight loss at approximate 120 °C, as the solvents began to evaporate: this means that the reinforced MC-PE can be safely used in thin flexible Li-based polymer batteries up to 100 °C.

**Figure 7:**
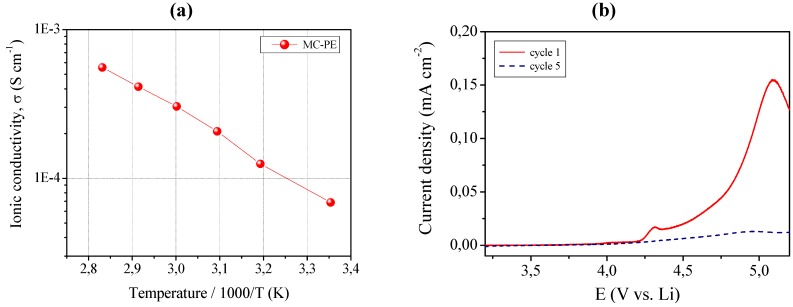
(**a**) Ionic conductivity *versus* temperature plot of the MC-PE reinforced quasi-solid polymer electrolyte. Data obtained by impedance spectroscopy; (**b**) Current *vs*. voltage curves at room temperature for MC-PE.

The ionic conductivity was evaluated by means of impedance spectroscopy and the Arrhenius plot is shown in Figure 7, left layer (a). MC-PE demonstrated an acceptable ionic conductivity of about 6.8 × 10^−5^ S cm^−1^ at ambient temperature. It increased with increasing the temperature, resulting in 5.5 × 10^−4^ S cm^−1^ at 80 °C. All impedance spectra obtained in the selected temperature range were found to be linear, with no sign of high-frequency semicircles which could indicate lack of gel homogeneity due to crystalline phase separation.

In addition to high ionic conductivity, sample MC-PE showed an appreciable anodic breakdown voltage, which makes it particularly valuable in view of a practical battery application. This is shown in Figure 7, right layer (b) which illustrates the current-voltage response of MC-PE obtained in the potential range between open circuit and 5.5 V *vs*. Li at ambient temperature by linear sweep voltammetry. The plateau was very flat and straight; this very low residual current level prior to breakdown voltage, with no peaks in the lower voltage range, confirmed the high purity of the prepared sample and the synthesizing method adopted, because the system as a whole is sensitive to oxygen, water and other impurities. The increase of the current during anodic scan, which is related the decomposition of the electrolyte, was taken in correspondence to the onset of a low current peak at approximate 4.4 V *vs*. Li. Nevertheless, if such low peak is not connected to the reaction of the electrolyte, then the stability range can be extended at least to 4.7 V *vs*. Li, a really interesting value. Tests are being carried out to deepen this aspect.

**Figure 8:**
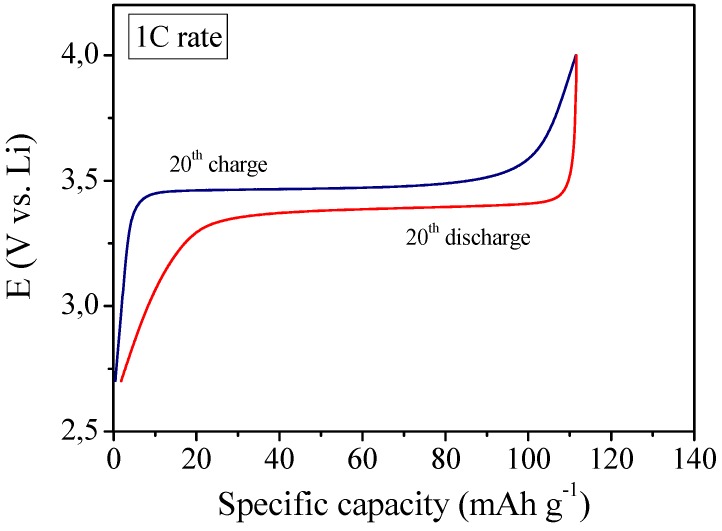
Ambient temperature galvanostatic charge-discharge profiles of the lithium polymer cell assembled by sandwiching the MC-PE reinforced polymer electrolyte between a LiFePO_4_/C cathode and a Li metal anode, at 1C current rate.

In view of the possible practical application of the MC-PE reinforced polymer electrolyte, a laboratory scale Li cell was assembled by combining a lithium metal anode with a LiFePO_4_/C cathode and using MC-PE as the electrolyte separator. Its electrochemical behavior was investigated by means of galvanostatic charge/discharge cycling at ambient temperature. The first results of galvanostatic cycling were found to be very interesting as depicted in Figure 8 which shows the 20th galvanostatic charge and discharge profiles at 1C current regime. In fact, at 1C rate the cell was able to deliver a specific capacity of about 110 mAh g^-1^, that is only about 12% lower with respect to the previously discussed not reinforced RC-1 polymer electrolyte membrane (see Figure 5 at the same 1C current rate for comparison). Good performance at high current rate might be ascribed to the efficient ionic conduction in the polymer separator and the favorable interfacial charge transport between electrodes and electrolyte in the cell. The potential drop between the charge and discharge plateaus was small, which means low resistance of the cell. Moreover, the Coulombic efficiency was found to be almost 100%, thus indicating a good interfacial behavior between electrodes and reinforced polymer electrolyte during the charge/discharge cycles of the cell.

### 2.3. Composite Quasi-Solid Polymer Electrolyte Membranes Reinforced By Nanoscale Microfibrillated Cellulose Fibers

As demonstrated in the previous paragraph, the use of cellulose handsheets as a reinforcement for quasi-solid polymer electrolytes led to a remarkable increase of the tensile resistance, Young’s modulus and handling ability of the membranes but, on the other hand, caused a slight decrease of the ionic conductivity and overall performance in lithium cell. A cellulose handsheets consists of a matted of felted sheet of fibers, therefore the use of such a reinforcement clearly reduced the possibility of ionic conduction inside the membrane structure. 

On the contrary, by using nanoscale microfibrillated cellulose (MFC) fibers this problem could be overcome without losing the positive impact of cellulose on the mechanical properties. MFC fibers consist of cellulosic individual nanoparticles with a lateral dimension of around 5 nm, which normally aggregate thus forming particles with lateral dimensions between 10 and 30 nm, or even higher. In length they can achieve few microns. MFC can form a highly entangled network with outstanding mechanical properties [26]. Thus, the incorporation of MFCs as reinforcement was here proposed in order to combine the good ionic conductivity of the neat quasi-solid photo-polymerized polymer electrolytes with the excellent mechanical behavior of the reinforced membranes. The appearance of the composite quasi-solid polymer electrolyte, namely MFC-PE, obtained with the incorporation of 4 wt.% of MFCs, is shown in Figure 1c. The composition of the polymeric matrix was the same as for the previously discussed RC-1 sample and the preparation method is discussed in Paragraph 3.4.

**Figure 9:**
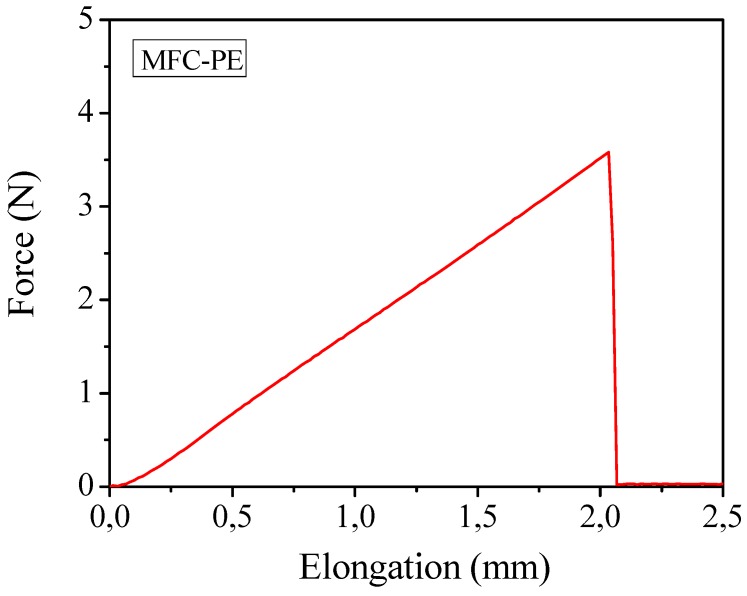
Mechanical measurements through traction test of the reinforced MFC-PE at ambient temperature.

Mechanical properties obtained by means of tensile test studies on the polymer composites containing the liquid electrolyte (a typical force-elongation curve is reported in Figure 9) gave an average Young’s modulus of about 32 MPa and an average tensile strength of approximate 2.0 MPa. The results were appreciable for the envisaged applications and more than satisfactory if considered that, in this case, the percentage of cellulose present in the membrane was highly reduced (from 10%–15% in the case of the previously discussed MC-PE to 4% of this MFC-PE). The outstanding handling ability, flexibility and bending endurance of the electrolyte membrane were proved also by means of bending test. To the purpose, sample MFC-PE was rolled around cylinders of diameter ranging between 32 and 3 mm and no sign of cracking was observed even when rolled around the cylinder with the lower diameter. Also in this case, *i.e.*, by using MFCs as reinforcing agent, such an excellent mechanical behavior may also help in improving the safety features of the PE membranes.

The thermal stability of the MFC-PE sample was assessed by thermo-gravimetric analysis under nitrogen. A distinct weight loss at approximate 110 °C was observed, as the solvents began to evaporate, meaning that the sample can be safely used up that temperature value.

**Figure 10:**
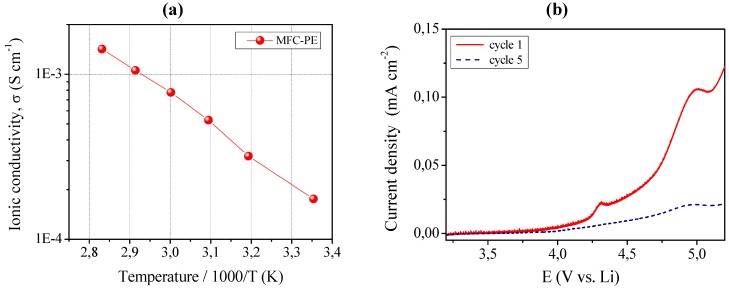
(**a**) Conductivity *versus* temperature plot of the MFC-PE reinforced quasi-solid polymer electrolyte. Data obtained by impedance spectroscopy; (**b**) Current *vs*. voltage curves at ambient temperature for MFC-PE.

The ionic conductivity was evaluated by impedance spectroscopy and the Arrhenius plot is shown in Figure 10, left layer (a). MFC-PE demonstrated an ionic conductivity of about 1.8 × 10^−4^ S cm^−1^ at ambient temperature, which increased at 80 °C resulting in the very interesting value of 1.4 × 10^−3^ S cm^−1^. Also in this case, all the impedance spectra obtained in the selected temperature range were found to be linear, with no sign of high-frequency semicircles. Such a reinforced quasi-solid polymer electrolyte membrane presented high ionic conductivity values, in fact almost comparable to those of RC-1 sample (see Figure 3 for comparison).

MFC-PE also showed an appreciable anodic breakdown voltage, as shown in Figure 10 left layer (b), which illustrates the current-voltage response obtained in the potential range between open circuit and 5.2 V *vs*. Li at ambient temperature by linear sweep voltammetry. The plateau was observed to be flat and straight; this very low residual current level prior to breakdown voltage, with no peaks in the lower voltage range, confirmed the high purity of the prepared sample and the synthesizing method adopted which assured the complete removal of water despite the water-based MFC suspension used. As already discussed for sample MC-PE, the increase of the current during anodic scan, which is related to the decomposition of the electrolyte, was taken at the onset of a low current peak at approximate 4.3 V *vs*. Li. If such low peak is not connected to the reaction of the electrolyte, then the stability range can be extended at least to 4.8 V *vs*. Li, a really interesting value.

**Figure 11:**
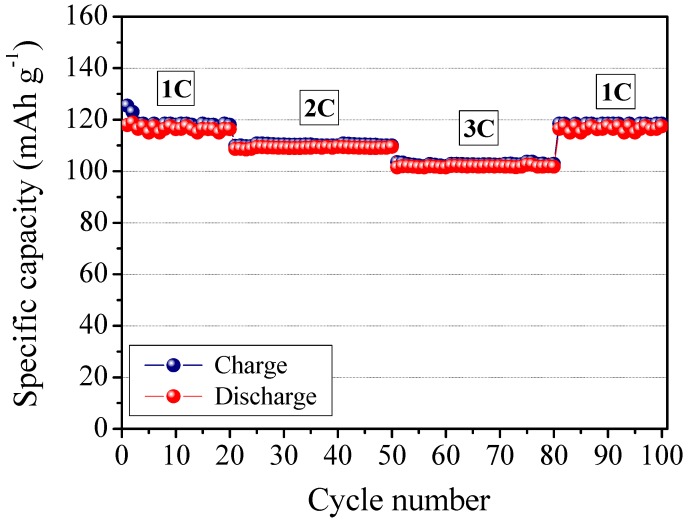
Ambient temperature cycling performance of a LiFePO_4_/MFC-PE/Li polymer cell, at different C-rates, from 1C to 3C.

Finally, the MFC-PE composite polymer electrolyte membrane was assembled in a complete lithium polymer cell laboratory prototype using lithium metal as the anode with a LiFePO_4_/C composite cathode, and its electrochemical behavior was investigated by means of galvanostatic charge/discharge cycling. The response of the prototype is reported in Figure 11. It shows the specific capacity of the cell as a function of the cycle number at ambient temperature and at different C-rates (*i.e.*, 1C, 2C, 3C). The cell delivers an average specific discharge capacity of about 120 mAh g^−1^ during the initial cycles, at 1C current rate. As the current density increased, the specific capacity decreased only slightly. Actually, at a high discharge rate of 3C, the cell was able to deliver an average discharge capacity higher than 100 mAh g^−1^. That is comparable to the results obtained with the not reinforced RC-1 sample.

Good performance at high current rate might be ascribed to the efficient ionic conduction in the polymer electrolyte separator and the favorable charge transport at the interface between the electrodes and the electrolyte in the cell. The potential drop in passing from charge to discharge was observed to be small indicating a low resistance of the cell and high Coulombic efficiency (*i.e.*, approaching 100%). The specific capacity remained highly stable during the whole cycling test, with no sign of abrupt decay, indicating that the presence of cellulose fibers does not affect the cycling ability of the assembled polymer cell. Finally, it is important to note that the system behaves correctly, with no abnormal drift even at high regimes; in fact, reducing the C-rate completely restores the specific capacity (see, in Figure 11, the specific capacity values from 3C to 1C after the 60th cycle). The results here demonstrate the feasibility of the MFC reinforced polymer electrolyte as a new electrolyte for advanced lithium polymer batteries.

## 3. Experimental Section

### 3.1. Materials and Methods

The materials used for the preparation of the quasi-solid polymer electrolyte membrane included:

1. Bisphenol A ethoxylate (15 EO/phenol) dimethacrylate (BEMA, M_n_: 1700, Aldrich, St. Louis, MO, USA);
2. Poly(ethylene glycol) methyl ether methacrylate (PEGMA-475, M_n_: 475, Aldrich, St. Louis, MO, USA);
3. Lithium bis(trifluoromethane) sulfonimide (LiTFSI, battery grade, FERRO Corp., Mayfield Heights, OH, USA);
4. Ethylene carbonate (EC, Aldrich, St. Louis, MO, USA) and diethyl carbonate (DEC, Aldrich, St. Louis, MO, USA).

The photo-initiator was 2-hydroxy-2-methyl-1-phenyl-1-propanone (Darocur 1173, Ciba Specialty Chemicals, San Ramon, CA, USA). 

The materials used as electrodes included an anode made of pure lithium metal foil (Chemetall, Frankfurt, Germany) and a LiFePO_4_/C disk composite cathode. The latter one was prepared in the form of a thin film by the so-called “doctor blade” technique, *i.e.*, casting on an Al current collector a *N*-methyl-2-pyrrolidone (NMP, Aldrich, St. Louis, MO, USA) slurry of the LiFePO_4_ active material (82 wt.%, typically about 3 mg cm-2) with acetylene black as electronic conducting additive (10 wt.%, Shawinigan Black AB50, Chevron Corp., San Ramon, CA, USA) and poly(vinylidene fluoride) as binder (8 wt.%, PVdF, Solvay Solef 6020, Solvay Solexis Inc., West Deptford, NJ, USA). High surface area nano-structured LiFePO_4_/C was obtained by a mild hydrothermal synthesis as reported by Meligrana *et al*. [27,28].

### 3.2. UV-Induced Free Radical Photo-Polymerization Process and Quasi-Solid Polymer Electrolyte Membrane Preparation

The preparation of the quasi-solid polymer electrolyte membrane (namely, RC-1) involved the use of a reactive mixture prepared as follows: the dimethacrylate (BEMA) to reactive diluent (PEGMA-475) ratio was kept 70:30, a 1.5 M LiTFSI in 1:1 EC DEC solution was added in the 45 weight percentage, along with 3 wt.% of free radical photo-initiator Darocur 1173. The mixture was later drawn into a film by coating it onto a polypropylene (PP) sheet with a calibrated, wire-wound applicator to obtain a thickness of about 200 μm. The process was carried out inside an Ar-filled dry glove box and the coated sheet was transferred into a UV transparent sealed box. This set up was then exposed to UV-irradiation till quantitative conversion of the double bonds was reached. The photochemical curing was performed using a medium vapor pressure Hg lamp (Helios Italquartz, Milan, Italy), with a radiation intensity on the surface of the sample of 32 mW cm^−2^. After the UV exposure, the box was taken back inside the glove box. Finally, a transparent, flexible and easy to handle film was obtained by peeling off from the PP sheet.

Before their use, the reactive monomers were kept open in the inert atmosphere of an Ar-filled dry glove box (MBraun Labstar, Garching, Germany, O_2_ and H_2_O content < 0.1 ppm) and also treated with molecular sieves (Molecular sieves, beads 4 Å, 8–12 mesh, Aldrich, St. Louis, MO, USA) to assure the complete removal of traces of water, as they may create problems in long-term properties when these membranes are considered as electrolytes in rechargeable Li-based batteries.

### 3.3. Cellulose Hand-Sheets Preparation, Modification and Reinforced Polymer Electrolyte Preparation

Kraft pulp, bleached and refined at 35 °SR (Schopper-Riegler degree [29]), was used as a raw material for hand-sheets preparation. A hard-wood (HW) to soft-wood (SW) combination of 60:40 w/w was re-pulped and blended using a high speed blender and made into desired concentration of 3 g L^−1^. It was later made to a suspension of 1.5 g L^−1^ which was introduced into a retention sheet-former (FRET) of 1500 RPM. The filtrate on copper wires was then dried at 90 °C under high vacuum to give a hand-sheet of 1.5 g with an average thickness of 100 µm. The handsheet with a HW:SW ratio of 60:40 was selected as reinforcing agent to be used in the composite polymer electrolyte membranes because it represented the best compromise between mechanical properties and ionic movement [30].

The hand-sheets were modified by photo-grafting technique using PEGMA-475 and benzophenone (Aldrich, St. Louis, MO, USA) as photo-initiator. In this grafting procedure, derived from Stachowiak *et al*. [31], the paper was swelled into a solution of benzophenone in ethanol. The swelled paper was UV irradiated for approximate 1 min under N_2_ flux and, then, dried at 60 °C. This pre-treated paper was swelled into a PEGMA/ethanol solution and, soon after, irradiated under flowing nitrogen. After washing in ethanol, the obtained modified cellulose was then dried under high vacuum at 120 °C to assure the complete removal of water and ethanol.

The obtained PEGMA-475 grafted cellulose hand-sheet was swelled into a reactive mixture (*i.e.*, RC-1, whose composition is described in Table 1) for 30 min and exposed to UV light for 3 min in 2 steps to obtain the cellulose-reinforced quasi-solid polymer electrolyte MC-PE. 

### 3.4. Nanoscale Microfibrillated Cellulose Fibers Preparation and Reinforced Composite Quasi-solid Polymer Electrolyte Preparation

MFC particles were prepared by treating bleached cellulose fibers at high pressure in a microfluidizer processor (model M-110 EH-30 by Microfluidics, Newton, MA, USA.) with a 400 and 200 µm diameter chamber. Cycles were varied in order to optimize the fibrillation process [32]. A MFC aqueous suspension (1 wt.%) was prepared.

The reactive formulation for the preparation of the reinforced quasi-solid polymer electrolyte membrane (MFC-PE) was based on the RC-1 formulation. The MFC suspension was added to the reactive formulation in order to obtain a composite containing 4 wt.% of fibers. This MFC-added liquid mixture was left 12 hours in oven at 60 °C to obtain the water evaporation and, subsequently, UV irradiated for 3 min. under N_2_ flux. Later, free, self-standing films were peeled off from the glass plates and treated in vacuum at 70 °C overnight.

### 3.5. Analyses and Characterization Techniques

The kinetics of the photo-polymerization process was investigated by using FT-IR spectroscopy (NICOLET-5700 FT-IR instrument by Thermo Fisher Scientific Inc., Illkirch, France) which collects the spectra in real time while the sample is irradiated by UV light), following the decrease in the area of the band at 1630 cm^−1^. The tests were carried out at ambient temperature on a UV transparent SiC wafer. The UV lamp used was Lightning curve LC-8 with an intensity of 15–16 mW cm^−2^.

Thermo-gravimetric analyses were done in the temperature range 25-600 °C using a TGA/SDTA-851 instrument (METTLER, Zurich, Switzerland) under N_2_ flux at a heating rate of 10 °C min^-1^. Differential scanning calorimetry (DSC) measurements were performed with a DSC-30 instrument (METTLER, Zurich, Switzerland) equipped with a low temperature probe. The temperature was increased from −140 to 100 °C at a heating rate of 10 °C min^−1^.

Mechanical measurements on the reinforced polymer electrolytes were carried out through tensile experiments according to ASTM Standard D638, using a Sintech 10/D instrument equipped with an electromechanical extensometer (clip gauge). At least five specimens for each sample were tested. The standard deviation in Young’s modulus (E) was 5%. Mechanical properties were evaluated by a bending test for which the membranes were rolled up around cylindrical hoses with radii ranging between 3 and 32 mm. 

The ionic conductivity of the quasi-solid polymer electrolytes at different temperatures was determined by Electrochemical Impedance Spectroscopy, namely EIS, frequency range from 1 Hz to 100 KHz at open circuit potential) using a PARSTAT-2273 potentiostat/galvanostat/F.R.A. (Frequency Response Analyzer) instrument from Princeton Applied Research (Oak Ridge, TN, USA.). The interfacial properties of the polymer electrolytes with respect to lithium metal as a function of contact time were measured by monitoring the time evolution of the impedance response of a symmetrical cell, formed by sandwiching the given sample between two lithium metal electrodes. The cells were stored at ambient temperature under open circuit conditions. This test was run by using the PARSTAT-2273 instrument. 

The anodic break-down voltage of the polymer electrolytes was evaluated by running a sweep voltammetry. Acetylene black over Al current collector and Li metal were the working and counter electrodes respectively. Potential scan range: from the open circuit potential to 6.0 V *vs*. Li; potential scan rate: 0.100 mV sec^−1^. Under these conditions, the onset of the current was assumed to indicate the decomposition voltage of the electrolyte membranes [17]. 

The laboratory-scale lithium polymer cells were assembled by arranging in sequence a lithium metal anode foil, a layer of the quasi-solid polymer electrolyte and a LiFePO_4_/C composite cathode film. The electrodes/electrolyte assembly was housed in a coffee bag envelope and, successively, sealed by hot pressing at about 100 °C. The electrochemical active area of the laminated cells was approximate 25 cm^2^. The Li polymer cells had a LiFePO_4_/C active material mass loading of about 4.5 mg cm^-2^. Both electrode fabrication and assembly of the cells were performed in an environmentally controlled dry room (10 m^2^, R.H. < 2% ± 1 at 20 °C, produced by SOIMAR, Caluso, Italy). The charge/discharge galvanostatic curves at different current regimes, rate capability and cycle life were obtained at ambient temperature using an Arbin Instrument Testing System model BT-2000.

## 4. Conclusions

In summary, we prepared high performing quasi-solid polymer electrolyte membranes for Li-batteries applications by UV-induced free radical photo-polymerization, which has proven to be a very advantageous technique due to its ease and rapidity in processing. The obtained polymer electrolytes showed wide electrochemical stability windows and very high ionic conductivity values even at ambient temperature. Furthermore, the mechanical properties and the ionic conductivity of the methacrylic-based quasi-solid polymer electrolytes reinforced with both cellulose handsheets and nanoscale microfibrillated cellulose fibers were excellent, demonstrating good overall electrochemical performance, intrinsic safety, eco-compatibility, low production cost and industrialization potential. Additionally, the prepared materials have shown excellent Li-electrolyte interfacial stability, cycling durability and Coulombic efficiency.

These results lead us to conclude that these kinds of polymer electrolyte membranes are very promising for Li-based batteries envisaged for high-end applications (e.g., automotive and aerospace) and encourage us to undertake further extensive experimental investigations.
